# Time Use Implication of Clean Cookstoves in Rural Settings in Ghana: A Time Use Study

**DOI:** 10.3390/ijerph18010166

**Published:** 2020-12-29

**Authors:** Rebecca Kyerewaa Dwommoh Prah, Daniel Carrion, Felix Boakye Oppong, Theresa Tawiah, Mohammed Nuhu Mujtaba, Stephaney Gyaase, Adolphine Kwarteng, Kenneth Ayuurebobi Ae-Ngibise, Oscar Agyei, Mieks Twumasi, Francis Agbokey, Kwaku Poku Asante, Darby W. Jack

**Affiliations:** 1 Kintampo Health Research Centre, P. O. Box 200 Kintampo, Ghana; atomistic4u@gmail.com (F.B.O.); theresa.tawiah@kintampo-hrc.org (T.T.); mohammed.mujtaba@kintampo-hrc.org (M.N.M.); stephaney.gyaase@kintampo-hrc.org (S.G.); adolphine.kwarteng@kintampo-hrc.org (A.K.); kenneth.ae-ngibise@kintampo-hrc.org (K.A.A.-N.); oscar.agyei@kintampo-hrc.org (O.A.); mieks.twumasi@kintampo-hrc.org (M.T.); francis.agbokey@kintampo-hrc.org (F.A.); kwakupoku.asante@kintampo-hrc.org (K.P.A.); 2 Department of Environmental Medicine and Public Health, Icahn School of Medicine at Mount Sinai, New York, NY 10029, USA; daniel.carrion@mssm.edu; 3 Department of Environmental Health Sciences, Mailman School of Public Health, Columbia University, New York, NY 10032, USA; dj2183@columbia.edu

**Keywords:** clean cookstoves, improved cookstoves, time use, firewood gathering time, cooking time, Ghana, rural, adoption

## Abstract

Whilst the health benefit of using clean cookstoves and fuels is widely known, there is limited information on the non-health benefit of these stoves, especially in low-middle-income countries. This paper reports the time use implications of using clean cookstoves and fuels by comparing liquified petroleum gas (LPG), an improved biomass cookstove (BioLite), and traditional biomass cookstoves (three-stone fires) in Ghana. Using survey-based time diaries, information on all the activities undertaken by study participants during a 24-h was collected and analyzed. The findings of the study show that LPG users spent significantly less time gathering firewood compared to the users of improved cookstoves and three-stone fires. LPG users spent slightly less time per cooking episode, generally, and there was no significant difference in cooking time across the three cookstoves mostly due to stove stacking. Time spent engaging in economic activities was highest for LPG users and improved biomass cookstove users, at least when compared to three-stone fire users. In this study, we provide evidence on the time use implications of clean cookstoves, highlighting their non-health benefits and supporting efforts towards the adoption and sustained used of clean cookstoves

## 1. Introduction

This paper investigates the time use implications of household energy interventions in the context of a randomized controlled trial in Ghana. It reports the difference in households’ time spent using clean cookstoves and fuels (liquified petroleum gas (LPG) and improved biomass cookstove (BioLite; BioLite Inc. Brooklyn, NY, USA)) compared to the use of traditional biomass cookstoves (three-stone fires). Household air pollution (HAP) remains a top priority public health problem, especially in developing countries. Globally, an estimated 2.8 billion people are exposed to HAP emanating from the use of solid fuels such as wood, crop residues, dung, and charcoal for cooking. About 3.8 million deaths globally and 739,000 deaths in Africa were attributable to HAP [1,2]. Exposure to HAP is also associated with increased risk of respiratory symptoms among pregnant women, low birth weight, stillbirth, preterm delivery, and impaired lung function [3,4,5,6,7,8]. In Ghana, HAP is one of the leading risk factors for disease burden attributable to about 14,458 deaths and about 8% of the disease burden in the country [9,10].

Switching to cleaner fuels for cooking is a viable public health solution to reduce HAP exposure and associated health problems, especially for women and children who have the highest exposure due to their household cooking roles [11,12]. Despite its benefits, adoption and sustained use of clean fuels is still limited. While the percentage of households relying on solid fuels is trending downward globally, the decline is generally slow in sub-Saharan Africa, where about 80% of the population use biomass fuels for cooking. Despite the decline in the proportion of households using solid fuels in Africa, the absolute number of households using solid fuels for cooking in Africa almost doubled between 1980 and 2010 due to increasing population during the same period [12,13]. In Ghana, about 74% of households, especially those in rural settings, use traditional biomass fuels for cooking whilst just about 18% (mostly in cities) use LPG for cooking [14]. Even when households have clean cookstoves and fuels, stove stacking (i.e., switching between clean cookstoves and other biomass traditional stoves) is still practiced for varied reasons, paramount of which are high costs of using clean cooking fuels and type of food cooked [12,15,16].

Whilst biomass fuels are free or low cost compared to cleaner fuels, the time spent gathering these fuels and cooking with traditional cookstoves is usually not considered. In rural settings where biomass fuels are mostly used, women and children are the prime collectors of these fuels for household use [12]. This consumes time that could be used for other productive activities [17,18]. Generally, households (mostly women and children) spend about 1.4 h a day gathering firewood [12]. In South Asia, women spend about 20 h a week gathering biomass fuels for cooking and about 4 h a day cooking with traditional biomass cookstoves [19]. The use of clean cook stoves is likely to reduce households’ firewood consumption, reduce the time used for firewood collection and cooking, and improve the lives of women and children by reducing the burden of these activities [12]. Evidence from South Asia shows that improved cookstoves reduced firewood consumption by 28.1%, firewood gathering time by 70 h a year, and cooking time by 1 h and 10 min per day [19]. 

Studying the impact of clean cookstoves on time allocation provides an opportunity to quantify the non-health benefits of the stoves that accrue in households. This may free household members time for other activities such as education, health, income generating, and socializing [12]. Better evidence on the time use implications of clean cookstoves may also support efforts towards the adoption of clean cookstoves and the attainment of universal access to clean cooking, which together contributes to the fulfillment of ten of the sustainable development goals (SDGs) [12,20]. Despite this, data on the time allocation implications of clean cookstoves, especially in rural settings, is rarely available. Although nationally representative numbers may be available, these likely underestimate the time that rural women devote to firewood collection and cooking [21]. Additionally, time allocation differs among setting, as it depends on the economic and geographical conditions such as natural resources [17].

This study aimed at evaluating the time implication of using clean cookstoves (LPG and improved biomass cookstove) compared to traditional biomass cookstoves in rural settings in Ghana. It reports the difference in households’ time use for firewood gathering, cooking, economic activities, and caring for the household. The results of this study provides evidence on the non-health benefits of clean cookstoves and supports efforts towards the adoption and sustained used of clean cookstoves, especially in rural setting.

## 2. Materials and Methods 

### 2.1. Study Design and Data Collection

**Figure 1:**
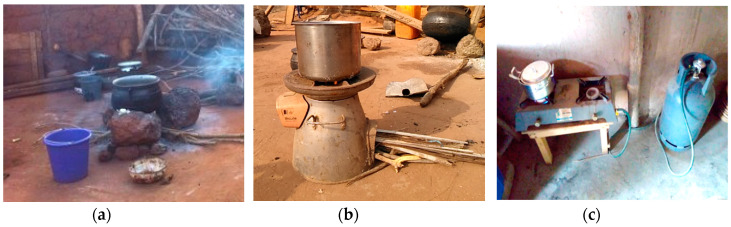
Three-stone (**a**), BioLite (**b**), and (**c**) liquefied petroleum gas (LPG) cookstoves [15].

This study draws from the Ghana randomized air pollution and health study (GRAPHS), which assessed whether clean cookstoves improve birth weight and reduce risk of pneumonia among infants [22]. Participants of GRAPHS were pregnant women in their first trimester, who were the primary cooks of their households and were non-smokers. They were randomized to either a control or one of two intervention groups. Participants in the intervention arms received either an improved biomass stove (BioLite arm) or an LPG cookstove and free supply of LPG throughout their enrollment in the study (LPG arm). BioLite cookstoves burn wood fuels but are designed for efficient combustion and heat transfer. They include a thermoelectric generator that powers a small fan, which increases airflow to the combustion chamber. Women in the control group continued to use their traditional cookstoves (three-stone fires). Figure 1 shows the three types of stoves used by participants of GRAPHS.

This paper reports the results of a time use survey conducted in 2015 and 2016. A survey-based time diary, adapted from the Ghana Statistical Service Time Use Survey [23] was administered to GRAPHS participants. Survey-based time diaries gather information about all activities undertaken by a respondent during a specific period, usually over the course of 24-h. This approach is commonly used in developing countries and provides a relatively accurate estimate of time use, especially with irregular activities [24,25]. Compared to other methods, it imposes less of a cognitive burden on respondents [26]. This is also the method used by the Ghana Statistical Service for their time use survey [23]. Trained fieldworkers asked study participants to recall the activities they undertook from the time they woke up until they went to bed the previous day. They then described in chronological order the activities they engaged in within those time bounds and indicated the approximate duration of each activity, starting with the first activity they did when they woke up. Particular attention was given to activities that were directly related to firewood gathering, cooking, income generation, and providing care for the family. Enumerators used local relevant reference periods to help participants recall their activities and times [26], e.g., listening to the local news at 6 a.m., 12 p.m., and 6 p.m. either on radio or a community information system and call to Muslim prayers at 5 a.m., 1 p.m., 3 p.m., 6 p.m., and 7 p.m. are common practices. Enumerators therefore asked participants what they were doing before, during, and after these events to help participants recall their activities and time. The study used experienced enumerators familiar with the study setting, routines, and practices of the local people. They were trained to understand the purpose of the survey design, use simple, clear, and understandable language/phrases, and learn how to correctly use code activities. This helped minimize any confusion that would result from an incorrect response.

The time diary was piloted to adapt it to the local conditions and to ensure that the survey did not impose an undue burden on study participants. Questions that were not applicable to the local study setting were either removed or modified. Additional questions on time spent on cooking and firewood gathering were added to generate a detailed picture of time use related to household energy.

There were two rounds of data collection: during GRAPHS and eight months after GRAPHS, when intervention arm participants were expected to have either sustained use of the clean cookstoves or reverted to using their traditional cookstoves. After eight months, no participant was expected to still have stock of their last LPG supply from the study. Each round consisted of three successive surveys (each lasting three months) to account for within-household variation in time allocation. This allowed for both during-and-after comparisons, as well as comparisons across study arms. The first round of data was collected from September 2015 to January 2016 and the second round of data was collected from September 2016 to December 2016.

For the purpose of this paper, the period during GRAPHS is referred to as the intervention period and the period after GRAPHS is referred to as the post-intervention period. 

### 2.2. Study Area

The study was conducted in the Kintampo North Municipality and Kintampo South District in the Bono East Region of Ghana. This is a mostly rural area with a population size of about 176,500 [14]. The study area lies within the Savannah ecological zone in the north and the Forest ecological zone in the south of the country. Firewood is abundantly available throughout the study area. Ghana has an average firewood consumption of about 92.8 kg/household/year and demand for firewood is expected to increase to more than 50 metric tons by 2020 [17,27,28,29,30,31,32,33,34]. Three-stone fires are the cookstoves mostly used by women in the area with wood being the primary source of fuel [28]. There are two major seasons in Ghana: the dry season (from December to March) and wet season (from April to November). Whilst cooking with LPG is mostly done in enclosed areas or covered kitchens, cooking with firewood (three-stone fires) is mostly done outdoors during the dry season and enclosed areas or covered kitchens during the wet season. Farming is the main occupation of the people in our study setting. The farming season in the study area runs from April to November during which time farmers would be planting or harvesting their crops. 

Ghana’s energy policy plan seeks to promote clean energy fuels for cooking. An example of such policy is the rural LPG program that seeks to promote LPG use in rural communities [16,30]. 

### 2.3. Data Analysis

Cleaned data were analyzed using Stata version 14.0 (Stata-Corp, College Station, TX, USA). Principal component analysis was used to compute an overall wealth index for each study participant based on assets ownership [31,32]. Study participants were grouped by their wealth indices into wealth quintiles namely: least poor, less poor, poor, more poor, and most poor. The socio-demographic characteristics of the study participants including education, marital status, employment status, religion, and wealth index were described using frequencies and percentages. 

For each study participant, the average time spent cooking, gathering firewood, income generation, and providing caregiving services to household members was computed. The computation was done separately for the intervention period (when participants were enrolled in GRAPHS) as well as the post-intervention period (when they had exited GRAPHS). Wilcoxon signed rank test was used to test for differences in cooking time, firewood gathering time, income generation, and caregiving time at these two time points (intervention and post-intervention periods). This was done for study participants in each of the study arms (control, BioLite and LPG). 

Additionally, for each of the time variables of interest—cooking time, firewood gathering time, income generating time, and caregiving time—the Kruskal–Wallis test was used to compare the three study arms. This was followed by a pairwise comparison between the three study arms using Dunn’s test [33]. Two different analysis namely, an intention-to-treat (ITT) analysis and as-treated analysis were conducted for cooking time. In the latter approach, time is computed based on the actual intervention used in cooking, whereas the former is based on the actual intervention assigned to study participants. Using similar statistical tests, the average number of simultaneous activities performed while cooking and the average number of cooking times (episodes) were compared between the three study arms. We defined cooking episode as the number of times a person cooked in 24 h. This was done using measurements obtained during the intervention and post-intervention periods. Moreover, an intervention and post-intervention period comparison of the average number of simultaneous activities performed while cooking, the average number of cooking times, and average time spent per cooking episode was performed for participants within each study arm using the Wilcoxon signed rank test. For each participant, time spent per cooking episode was estimated as the total number of minutes spent cooking per 24 h divided by the total number of cooking episodes per 24 h.

Results are presented as mean and 95% confidence interval. Test results were considered to be statistically significant if their corresponding *p*-values were < 0.05.

### 2.4. Ethics 

This study was approved by the Kintampo Health Research Centre Ethics Review Committee with reference number (KHRCIEC/2015-6). Written consent was obtained from all study participants before their participation in the study.

## 3. Results

A total of 539 women were enrolled–199 in the control, 197 in the BioLite and 143 in the LPG arm. The analysis, however, was restricted to 443 (82.2%) (control = 160, BioLite = 157, LPG = 126) participants who completed all rounds and surveys. The remaining 96 (17.8%) participants either moved out or were missed in at least one of the follow-up surveys after the initial survey. Table 1 presents the baseline description of the study participants. There were only minor differences in the characteristics of the participants across the different study arms. For all study participants, about 61% had some form of formal education, about 75% were employed (mostly in agriculture), and about 62% were married. These were similar across all three arms (Table 1). The ages of the study participants ranged between 15 and 47 years with a mean of 28 years. The overall average household size was 7 members across all three arms, with a corresponding average of 7 members for each of the study arms. The minimum household size was two for each of the clusters, while the maximum household size was 27, 15, and 25 for the control, BioLite, and LPG arms respectively. 

Stove stacking (the practice of switching between different cookstoves) was commonly practiced by participants in the intervention arms (LPG and BioLite arms) and this was observed at both time points. During the intervention period, 29 (18.47%) and 11 (8.73%) participants in the BioLite and LPG arms, respectively, used their traditional three-stone fires for cooking instead of their allocated cookstoves. This increased in the post-intervention period with 152 (96.82%) of participants in the BioLite arm and 123 (97.62%) of those in the LPG using their traditional stoves instead of their allocated intervention stoves. 

### 3.1. Firewood Gathering 

Time spent gathering firewood by participants at the two time points (intervention and post-intervention periods) was assessed. During the intervention period, when participants were receiving free LPG supply, participants in the LPG arm spent 8.53 min per 24 h gathering firewood whilst those in the BioLite and control arms spent significantly more time gathering firewood: 23.20 min and 27.59 min, respectively (*p*-value = 0.002) (Table 2). 

During the post-intervention period when there was no free supply of LPG, time spent gathering firewood significantly increased by 3.54 min for participants in the LPG arm (*p*-value = 0.022). However, for participants of the BioLite and LPG arms, there was a significant change in firewood gathering time during and after the intervention period (Table 2).

**Figure 2:**
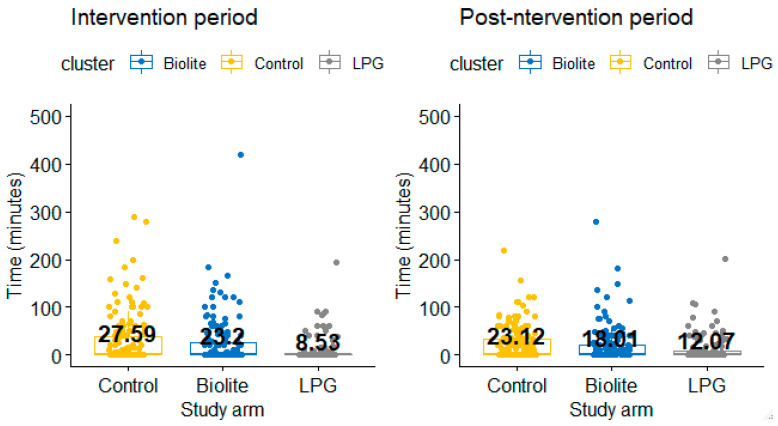
Difference in firewood gathering time between the study arms.

Compared across arms, both time point participants in the LPG arm spent significantly less time gathering firewood compared to participants in the BioLite and control arms at both time points (Figure 2).

### 3.2. Cooking Analysis 

#### 3.2.1. Average Cooking Time per 24 h: Intention to Treat Analysis

With the intention to treat analysis, average cooking time for all three arms ranged between 143.54 min (2 h·24 min) and 176.39 min (2h 56 min) per day. Among participants of the control arm, more time was spent on cooking during the intervention period than the post-intervention period (Table 3). As presented in Table 3, even though a similar trend was found in the BioLite and LPG arms, the difference in cooking time was not statistically significant. Compared across arms, there was no significant difference in cooking time between the study arms at both time points. 

#### 3.2.2. Average Cooking Time per 24 h: As Treated Analysis

Results from the ‘as treated’ analysis for the intervention period were not very different from the results of the ‘intention to treat’ analysis at the same time point (Table 3). There was, however, a significant reduction in cooking time for participants in the LPG and BioLite arms during the post-intervention period. LPG users used an average of 41.60 min per 24 h cooking with LPG, while the BioLite users spent an average of 35.65 min per 24 h for cooking with BioLite. 

#### 3.2.3. Cooking Episodes and Average Time per Cooking Episode

Overall, participants across the three study arms cooked more than 3 times a day. Participants in the BioLite arm cooked more times during the intervention period than in the post-intervention period (4.54 times vs. 3.86 times, *p*-value = 0.032). Similarly, participants in the LPG arm cooked more times during the intervention period than in the post-intervention period (4.47 times vs. 3.74 times, *p*-value = 0.018). However, for participants in the control arm, there was no significant difference in the average number of cooking episodes during and after the intervention period: 3.84 times compared to 3.73 times, *p*-value = 0.497 (Table 3). Moreover, during the intervention period, participants in the BioLite and LPG arms cooked more times compared to participants in the control arm (LPG = 4.47 times; BioLite = 4.54 times, control = 3.84, *p*-value = 0.015). However, at the post-intervention period, there was no significant difference in the number of cooking episodes for participants in the LPG, BioLite and control arm (Table 3).

During the intervention period, participants in the LPG and BioLite arms spent 39.07 min and 39.44 min per cooking episode respectively whereas those in the control arm spent a higher time of 42.95 min. Compared to the intervention period, post-intervention, participants in the control arm spent 2.52 min less per cooking episode (42.95 vs. 40.38, *p*-value = 0.001). For participants in the LPG arm, time spent per cooking episode was higher post-intervention: 42.24 min compared to 39.07, *p*-value = 0.037. During the intervention and post-intervention period, there was no difference in the time spent per cooking episode for participant in the BioLite arm (Table 3). The difference in time use per cooking episode across the study arms was statistically significant during the intervention period (lower for the LPG and BioLite arms compared to the control arm, *p*-value = 0.017), and there was no significant difference in time use in the post-intervention period (*p*-value = 0.277).

#### 3.2.4. Simultaneous Activities during Cooking

Participants reported of engaging in simultaneous activities during cooking. During the intervention period, participants in the control arm engaged in an average of 2.24 activities while those in the LPG and BioLite arms engaged in 1.92 and 1.85 simultaneous activities, respectively. The average number of simultaneous activities significantly reduced for all three study arms in the post-intervention period. At both time points, there was no significant difference in the average number of simultaneous activities between the users of BioLite and LPG.

### 3.3. Other Time Uses

Time used for other activities such as engaging in income generating activities and providing care giving services for household members, including the elderly and children, were also analyzed. This gives an indication of the additional time participants used to provide care for their household members and for performing economic activities during and after the intervention.

#### 3.3.1. Time Spent on Income Generating Activities

**Figure 3:**
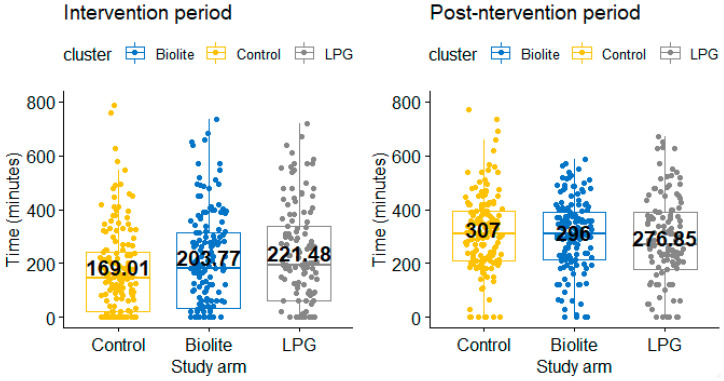
Difference in time spent engaging in income generating activities between study arms.

During the intervention period, participants in the LPG and BioLite arms spent 221.48 min and 203.77 min engaging in income generating activities per day, respectively. Those in the control arm spent a lesser time of 169.01 min. For the post-intervention period, time spent on income generating activities increased significantly by 55 min for participants in the LPG arm, 92 min for participants in the BioLite arm, and 138 min for participants in the control arm (Table 4). Compared across arms, during the intervention period, LPG users spent more time on income generating activities compared to participants who were in the control arm. However, there was no significant difference in time spent on income generating activities between the LPG and BioLite arms, as well as between the BioLite and control arms during the intervention period. There was no significant difference in time spent on income generating activities between the three study arms (Figure 3) in the post-intervention period. 

#### 3.3.2. Average Time Spent Providing Care to Household Members

**Figure 4:**
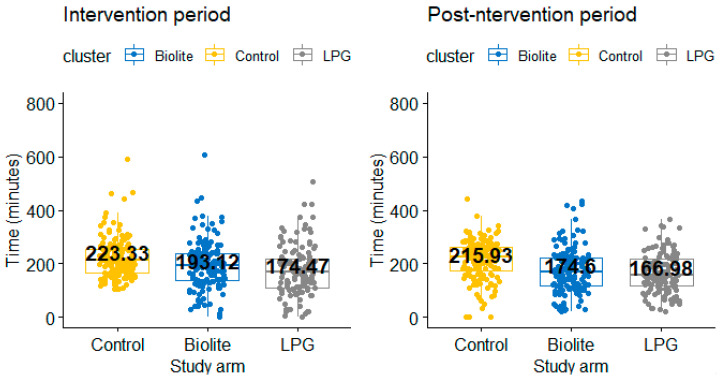
Difference in care-giving time between the study arms.

During the intervention period, participants in the BioLite and LPG arms spent less time providing care compared to those in the control arm(LPG arm = 174.47 min; BioLite arm = 193.12 min; control arm = 223.33 min; *p*-value < 0.001) (Figure 4). Similar results were observed during the post-intervention period, with participants in the control arm spending more time caring for household members compared to participants in the BioLite and LPG arm (Table 4). In each of the study arms, there was no significant difference in time spend caring for household member during and after the intervention period. 

## 4. Discussion

This study contributes to research regarding the time allocation implications of using clean cookstoves as compared to traditional biomass cookstoves in a rural African setting. It compares the differences in time used for firewood gathering, cooking, providing care for household members, and engaging in economic activities among users of LPG cookstoves, BioLite cookstoves, and three-stone fires. The results suggest that LPG cookstoves significantly reduce time spent gathering firewood compared to three-stone fire cooking and has the potential to reduce average cooking time per cooking episode and also free up time for income generating activities. This aligns with the results of studies conducted in South Asia (among female cooks in India, Bangladesh, and Nepal), which also showed that using improved cookstoves reduced firewood gathering and cooking time compared to using traditional biomass cookstoves [19]. 

Firewood gathering time was highest for participants in the control arm who used three-stone fires followed by those in the BioLite arm. In studies conducted in India, Bangladesh, and Nepal, households who used traditional cookstoves recorded higher firewood gathering time compared to households who used improved cookstoves [19]. 

The average number of minutes spent gathering firewood (maximum average of 27.5 min for the control arm and for the control) was comparable to that reported in Madagascar, where rural women spent an average of 27 min gathering firewood. Contrarily, in Benin, Ethiopia, and Tanzania, women spend less time gathering firewood because firewood gathering is mostly done by men and is part of paid work [34,35]. In our study setting, gathering firewood is mostly done by women. A time use survey conducted in Ghana shows that women spend more time (45 min) gathering firewood than men (25 min) [36].

Firewood gathering time was higher during the intervention period than the post-intervention period for both the BioLite and Control arms. This can be explained by the observed practice in our study setting where, in the days leading to delivery and after delivery, older relatives move in with the soon-to-be mother or new mother to provide post-natal care for the new mother and baby [37]. Increased number of people in the household requires more firewood for cooking and boiling water. Again, after delivery, firewood is needed for water boiling for the new mother and the baby. For most households, firewood is gathered for these purposes and this work is mostly done by the women. This practice also partly explains the higher cooking time in the intervention period when the women were either pregnant or new mothers than the post-intervention period, which was a year after delivery when the women had returned to their routine. It must be noted that even though women receive support from older relatives with their babies after delivery, many household chores like cooking and firewood gathering are done by the woman in the weeks prior to delivery and a few weeks after (usually after 40 days). 

The ‘intention to treat’ analysis of cooking time showed that participants in the LPG and BioLite arms used an average of 161.72 min and 165.87 min, respectively, to cook during the intervention period, and 155.51 min and 157.76 min, respectively, during the post-intervention period. Because some participants in the intervention arms were stove stacking as reported above, their cooking time may have been influenced by this practice. Thus, the ‘as treated’ analysis which considered only the time spent cooking with the allocated stove was conducted. This showed a lesser cooking time for participants in the intervention arms, especially during the post-intervention period (LPG = 41.60 min; BioLite = 35.65 min). Generally, cooking time in all three arms (with the exception of the as-treated analysis of the post-intervention period cooking time for the LPG arm and BioLite) was higher than the average cooking time reported by the Ghana time use survey, where females spent 98 min on food management (cooking and serving of meals). The Ghana time use survey did not offer details of which stove was used for cooking [36].

During the intervention period, while participants were still using their intervention cookstoves, participants in the intervention arms reportedly spent between 35 min (BioLite Arm) and 53 min (LPG arm) more engaging in economic activities than those in the control arm. This was contrary to the post-intervention period, where there was no significant difference in time used for economic activity across the study arms. This could mostly be because over 95% of the participants in the intervention arms stopped using their intervention stoves and were now using their traditional cookstoves. This also shows that clean cookstoves, especially LPG cookstoves, have the potential to save time for economic activities if used exclusively. 

The observed general trend in time use among the study participants across all three study arms suggests that women spend more time cooking and providing care for household members including children and the elderly during the intervention period than the post-intervention phase; this was true across all study arms. The opposite was true for time spent on income generating activities. This observed trend could be explained by the practice in the study setting, where pregnant women and new mothers spend little to no time engaging in income generating activities and rather spend time on in-house activities such as cooking and providing care for household members including their new babies. The intervention period was when the women were either pregnant or new mothers whilst the post-intervention period was a year or more after delivery when the women have returned to their routine. 

The women spending more time on unpaid activities (firewood gathering, cooking, providing care for household members) is a common occurrence for women in the country and other sub-Saharan African countries [34,35,38]. Women are not remunerated for these activities and thus their efforts towards these activities are not recognized as considerable contributions to the household income and national economy [39]. This contributes to poverty among women, especially those in rural settings. Women tend to have a limited role in household decision-making and control over major decisions, such as those concerning their health and choice of cooking fuels. Again, spending time on household responsibilities impedes women’s ability to attain higher education, advance in their careers, and earn higher income [40]. With higher educational attainment and income levels, women are more likely to adopt technologies that help with chores [39], such as LPG and electric cookstoves that cook faster and promote better health. Policies seeking to empower and enhance women’s wellbeing in society should consider interventions that promote widespread household adoption and sustained use of clean cookstoves such as LPG in rural settings. This will reduce time spent cooking and gathering firewood, the risk of firewood gathering, as well as the environmental and health impact of using biomass fuels. The results of this study afford the opportunity for further research into how women can be engaged to utilize the time saved from using these clean cookstoves in a way that promotes their economic wellbeing and development. 

Usually, the time spent by women on the various activities (firewood gathering, cooking, providing care for the household) are dictated by existing social norms and cultural behaviors, all of which impact the wellbeing of those who undertake these activities. It would be interesting to also know how social and cultural practices of the people affect cooking practices and time use in the study setting. 

Accurately recalling the activities and time spent is an innate limitation of time use surveys in developing countries [24], mostly because participants usually have limited literacy and may not enumerate the passage of time. This leads to over or underestimated duration of certain activities. Our study was designed in a way that enhance recall accuracy and adequately addresses the challenge of recall. The time diaries method used does not require respondents to be literate or numerate but rather requires them to be familiar with clock-oriented time [26]. Participants were asked to recall their activities in a chronological order which emulates autobiographical memory and thus enhance recall accuracy [26]. The 24 h reference period was short enough to allow participants to recall their activities. Participants who struggled to recall the exact activities and time were encouraged to give a narrative of their previous day’s activities whilst the enumerator took notes. This style of engaging participants made it easier for them to remember what happened [24]. Additionally, participants were also pre-informed about enumerators’ visits 24 to 48 h prior to their visits. To further help participants’ recall the time an event took place, enumerators used local relevant recall clues. For instance, listening to the local news at 6 a.m., 12 p.m., and 6 p.m. either on radio or community information system and call to Muslim prayers at 5 a.m., 1 p.m., 3 p.m., 6 p.m., and 7 p.m. are common practices. Enumerators therefore asked participants what they were doing before, during, and after these events to help participants recall their activities and time. The six repeated surveys helped check the precision of the reported time use while the time diary was used. This style of data collection has been used in similar settings and has provided accurate estimates for time use [24,25,36]. A future study could benefit from adopting an observer participation approach in addition to the time diary method. This could provide a more accurate and detailed description of the people’s daily routines and time used.

Another limitation of the study was the discontinued use of intervention stoves after the intervention phase of the study and stove stacking by some participants in the intervention arms. Even though participants in the LPG arm received a free supply of LPG during the intervention period, 8.73% of them reported using their three-stone fires and 97.62% switched to use their traditional three-stone fires during the post-intervention period. This explains the firewood gathering time recorded for participants in the LPG arm. Again, more than 90% of the study participants stopped using their intervention stoves. This mass discontinued use of LPG in the post-intervention period was mostly because the free supply of LPG stopped once women existed GRAPHS and they were expected to purchase LPG themselves. Several reasons have been cited by our study participants for the discontinued use of LPG and stove stacking. These include the cost of LPG, type of meal cooked, household size, and the preferred taste of some meals cooked on biomass stoves [15]. This discontinued use and stove stacking practice may have affected the study’s ability to assess the full and longer-term implications of using these stoves on time. Despite this, the study’s aim of providing information on the time implication of these stoves was achieved. It highlights the time implication of stove stacking and the potential time-gains from clean cookstoves stoves such as time saved from firewood gathering and cooking, as well as freed-up time for economically gainful activities. It also provides an opportunity for further research into households cooking and energy needs in order to eliminate stove stacking and enhance exclusive clean fuel use so as to achieve higher time gains from clean fuels that would be beneficial to households. It also calls for further research to understand household behavior with respect to clean cookstoves use. Research promoting the adoption and continued use of clean cookstoves is currently underway in the study setting. This study will provide an opportunity for researchers to further understand the factors influencing the adoption of clean cookstoves, as well as assess the longer term non-health effects of these clean cook stoves [38].

## 5. Conclusions

Our study aimed at assessing the time use implication of clean cookstoves on households in rural settings by comparing time spent by households who use clean fuels (LPG), biomass improved cookstoves (BioLite), and traditional biomass cookstoves (three-stone fires). Despite the widely known health benefits of clean cookstoves, not much is known about the non-health benefits, such as those evaluated in this study in sub-Saharan Africa. Using survey-based time diaries, information on all activities undertaken by study participants within a 24-h period were collected and analyzed. Our results showed that using LPG cookstoves reduced time spent on firewood gathering and had the potential to reduce cooking time per cooking episode, saving time for income generating economic activities. Stove stacking limited the time saved from using these stoves. The main limitation of the study was the discontinued use of the intervention stoves in the post-intervention phase of the study and stove stacking by some participants in the intervention arms. This impacted the estimation of the longer-term implication of using these stoves on time. Despite this, the study’s results provide an understanding and a clearer picture of the time burden on households, especially women, as a result of using biomass traditional cookstoves and the potential time gains from clean cookstoves. This information could inform policymakers and stakeholders on the need to promote these clean cookstoves especially in rural settings where firewood is the main fuel for cooking. It supports efforts towards the adoption and sustained use of clean cookstoves and calls for policies that promote clean cookstoves adoption in order to improve the health and wellbeing of households, especially women and children.

## Figures and Tables

**Table 1:** Description of study participants.

Variable	Overall (*n* = 443)		Control (*n* = 160)		BioLite (*n* = 157)		LPG (*n* = 126)	
	*n*	%	*n*	%	*n*	%	*n*	%
*Education*								
None	174	39.28	61	38.13	60	38.22	53	42.06
Primary school	133	30.02	43	26.88	60	38.22	30	23.81
Middle/JHS	123	27.77	51	31.87	33	21.02	39	30.95
Technical/Commercial/SHS	13	2.93	5	3.13	4	2.55	4	3.17
*Marital Status*								
Married	275	62.08	96	60.00	104	66.24	75	59.52
cohabitation	124	27.99	55	34.38	35	22.29	34	26.98
Divorced	1	0.23	1	0.63	0	0.00	0	0.00
unmarried	43	9.71	8	5.00	18	11.46	17	13.49
*Employment status*								
Unemployed	111	25.06	41	25.62	40	25.48	30	23.81
Employed	332	74.94	119	74.38	117	74.52	96	76.19
*Religion*								
Christian	275	62.08	109	68.13	95	60.51	71	56.35
Muslim	124	27.99	35	21.88	42	26.75	47	37.30
Other	11	2.48	2	1.25	6	3.82	3	2.38
None	33	7.45	14	8.75	14	8.92	5	3.97
*Wealth index*								
Least poor	107	24.15	49	30.63	37	23.57	21	16.67
Less poor	76	17.16	27	16.88	28	17.83	21	16.67
Poor	80	18.06	21	13.13	33	21.02	26	20.63
More poor	96	21.67	32	20.00	35	22.29	29	23.02
Poorest	84	18.96	31	19.38	24	15.29	29	23.02
*Age:* mean|Standard deviation	28.45	6.93	28.31	7.20	28.87	6.77	28.10	6.79
*Household size*								
Mean|min–max	6.59	2−27	7.44	2–27	6.08	2–15	6.13	2–25

The data on age is presented as mean and standard deviation.

**Table 2:** Average time spent on firewood gathering (paired analysis).

Study ARM	Intervention Period Mean (95% CI)	Post-Intervention Period Mean (95% CI)	(*p*-Value) ^§^
Control (*n*= 160)	27.59 (19.16–36.03)	23.12 (17.65–28.59)	0.653
BioLite (*n* = 157)	23.20 (15.34–31.06)	18.01 (12.13–23.89)	0.517
LPG (*n* = 126)	8.53 (4.02–13.05)	12.07 (7.17–16.97)	0.022
(*p*-value) ^ɠ^	0.002	0.025	

^§^*p*-value for difference in time spent during the intervention and post-intervention period. ^ɠ^*p*-value for difference in time between the study arms. Time spent is measured in minutes per a 24-h duration.

**Table 3:** Cooking analysis.

Study Arm	Intervention Period Mean (95% CI)	Post-Intervention Period Mean (95% CI)	(*p*-value) ^§^
Intention to treat analysis			
Control (*n* = 160)	159.38 (149.80–168.96)	150.22 (143.54–156.91)	0.037
BioLite (*n* = 157)	165.87 (155.35–176.39)	157.76 (150.13–165.40)	0.342
LPG (n = 126)	161.72 (149.83–173.62)	155.51 (146.72–164.31)	0.368
As treated analysis (time spent cooking on the assigned cookstove)			
Control (*n* = 160)	159.38 (149.80–168.96)	149.89 (143.16–156.62)	0.030
BioLite *(n* = 157)	159.99 (149.25–170.72)	35.65 (28.27–43.02)	<0.001
LPG (*n* = 126)	160.28 (148.25–172.31)	41.60 (33.91–49.29)	<0.001
Cooking episodes			
Control (*n* = 160)	3.84 (3.56- 4.12)	3.73(3.58- 3.89)	0.497
BioLite (*n* = 157)	4.54 (4.16–4.92)	3.86 (3.68–4.03)	0.032
LPG (*n* = 126)	4.47 (4.08- 4.87)	3.74 (3.53- 3.94)	0.018
Time spent per cooking episode			
Control (*n* = 160)	42.95 (41.33–44.57)	40.38 (39.33–41.43)	0.001
BioLite (*n* = 157)	39.44 (37.63–41.25)	41.16 (40.07–42.25)	0.276
LPG (*n* = 126)	39.07 (36.74–41.40)	42.24 (40.60–43.88)	0.037
Simultaneous activities during cooking			
Control (*n* = 160)	2.24 (2.04–2.45)	0.78 (0.69 -0.86)	<0.001
BioLite (*n* = 157)	1.85 (1.64–2.05)	1.03 (0.91–1.15)	<0.001
LPG (*n* = 126)	1.92 (1.65–2.18)	0.92 (0.82–1.02)	<0.001

^§^*p*-value for difference in time spent during the intervention and post-intervention period. Time spent is measured in minutes per a 24-h duration.

**Table 4:** Average time spent on non-cooking activities.

Study Arm	Intervention Period Mean (95% CI)	Post-Intervention Period Mean (95% CI)	(*p*-Value) ^§^
Average time spent on income generating activities			
Control (*n*= 160)	169.01 (144.15–193.88)	307.00 (283.94–330.05)	<0.001
BioLite (*n* = 157)	203.77 (175.46–232.07)	296.00 (273.30–318.71)	<0.001
LPG (*n* = 126)	221.48 (188.28–254.67)	276.85 (246.66- 307.03)	0.006
Average time spent caring for household members			
Control (*n* = 160)	223.34 (208.91–237.74)	215.93 (204.65–227.22)	0.621
BioLite (*n* = 157)	193.12 (179.07–207.17)	174.60 (161.77–187.43)	0.049
LPG (*n* = 126)	174.47 (157.62–191.33)	166.98 (153.94–180.01)	0.644

^§^*p*-value for difference in time spent during the intervention and post-intervention period. Time spent is measured in minutes per a 24-h duration.
